# Exosomes from miR-149-3p-transfected menstrual blood-derived mesenchymal stem cells ameliorate inflammation and migration of endometriosis cells 

**DOI:** 10.22038/ijbms.2025.86443.18677

**Published:** 2025

**Authors:** Hoda Fazaeli, Nasim Hayati Roodbari, Ehsan Ehsani, Azar Sheikholeslami

**Affiliations:** 1 Department of Biology, Faculty of Sciences and Converging Technologies, Science and Research Branch, Islamic Azad University, Tehran, Iran; 2 Department of Biology, Faculty of Sciences, Islamic Azad University, Roudehen Branch, Roudehen, Iran; 3 Department of Cell Biology and Regenerative Medicine, Academic Center for Education, Culture and Research (ACECR), Qom branch, Qom, Iran

**Keywords:** Endometriosis, Exosomes, Inflammation, Mesenchymal stem cells, MicroRNA 149, Migration

## Abstract

**Objective(s):**

Endometriosis carries remarkable social, public health, and financial consequences. Based on two theories of retrograde menstruation and stem cells, menstrual blood-derived stem cells (MenSCs) play a significant role in endometriosis since key genes of critical cellular processes are differentially expressed in the MenSCs of endometriosis and non-endometriosis women (E- and NE-MenSCs, respectively). In this study, E-MenSCs were isolated from the menstrual blood of women with various endometriosis subtypes. We tried to find the proper microRNA (miRNA) and assayed the effects of exosome-encapsulated miRNA on modulating the gene expression profile and functional pattern of E-MenSCs.

**Materials and Methods:**

After in silico selection of miR-149-3p using publicly accessible algorithm-based databases, E- and NE-MenSCs were cultured as controls, and the other experimental groups were as follows: E-MenSCs transfected with empty and miRNA vectors (E-MenSC+BB and E-MenSC+miR), and E-MenSCs treated with exosomes derived from non-transfected and miRNA-transfected NE-MenSCs (E-MenSC+Exo and E-MenSC+T-Exo). Then, the expression level of selected genes, the level of interleukins (ILs) and oxygen reactive species (ROS), the protein level of β-catenin and Ki-67, and the migratory ability were assessed through real-time PCR, ELISA, western blot, and scratching tests, respectively.

**Results:**

Although both E-MenSCs+T-Exo and E-MenSC+miR showed down-regulation of IL-6, -8, and -10, neither had decreased IL-1β, vascular endothelial growth factor, IDO1, and KRAS levels. Furthermore, only the IL-6 protein level was significantly decreased in the E-MenSC+miR group, but the levels of IL-6, IL-8, ROS, β-catenin, and Ki67 were significantly lower in the E-MenSCs+T-Exo group compared to the E-MenSCs.

**Conclusion:**

The potential of exosomes as miRNA carriers could be considered in developing novel endometriosis therapies.

## Introduction

The presence of endometrial-like tissue outside the uterus is referred to as endometriosis, which affects 5–10% of women in the world who are of reproductive age (1). After puberty, the risk of developing endometriosis is at its maximum due to oestrogens, the onset of menstruation, and sexual activity (2). Endometriosis’ health impact includes persistent pain and high lifelong costs (3). Unfortunately, a significant portion of women with endometriosis do not find current surgical and medicinal treatments safe and effective. Moreover, hormonal therapy, which is commonly used, is not suitable for those patients who wish to conceive. Thus, non-hormonal therapies are needed to improve outcomes while prioritizing patient well-being (4). On the other hand, it is still unknown exactly how endometriosis develops. According to the widely accepted theory of retrograde menstruation (RM), which was put forth by Sampson *et al*. in 1927 (5), endometrial glandular epithelial and stromal cells are transported in menstrual blood and enter the pelvic cavity through the fallopian tubes. These cells have the capacity to penetrate and develop near the pelvic peritoneum and ovaries, which can lead to the formation of pelvic endometriosis (5). Although the majority of women experience retrograde reflux, only 10–15% of them go on to develop endometriosis (6). The low prevalence of endometriosis in RM patients may be explained by the stem cell theory, since only a tiny fraction of them have endometrial stem cells with aberrant adhesion processes that cause them to invade the pelvic cavity and result in endometrial lesions (7). 

According to both theories of retrograde menstruation and stem cells, Menstrual blood is a key source of stromal stem cells, potentially linking endometrial lesions to endometriosis. The cellular composition of menstrual blood may play a significant role in the pathogenesis of endometriosis. Menstrual blood contains a population of adherent cells with regenerative potential known as endometrial regenerative cells or menstrual blood-derived stem cells (MenSCs) (8), which share some morphological and functional characteristics with mesenchymal stem cells (MSCs). These include the expression of the markers CD29, CD44, CD73, CD90, and CD105 as well as the absence of hematopoietic and endothelial markers (9). They can differentiate into various cell lineages (8). 

Extracellular vesicles (EVs), on the other hand, have been extensively documented as the primary paracrine signaling mechanism used in cell therapy (10). Based on their biogenesis, EVs are classified into three types: microvesicles, exosomes, and apoptotic bodies (11). They discovered that the active component in MSC-conditioned media is exosomes (12). Exosomes are nano-sized intraluminal vesicles found in multi-vesicular bodies (MVBs) and produced by different cell types after MVB fusion with the plasma membrane (13). They play an important role in cell-cell communication by transporting proteins, lipids, and nucleic acids (14). MSC-derived exosomes have recently received much interest due to their broad therapeutic effects on a variety of disorders (15).

Our recent findings suggest that MenSCs contribute to endometriosis etiology, with MenSCs of women with endometriosis (E-MenSCs) showing differential expression of genes related to inflammation, apoptosis, migration, and angiogenesis compared to MenSCs of non-endometriosis women (NE-MenSCs), promoting lesion development (16). Following that, we used exosomes secreted from NE-MenSCs to restore the dysregulation of the altered gene expression profile of E-MenSCs. This demonstrated encouraging results of exosome potential as a cell-free product in treating endometriosis (17) and can be considered as evidence of the role of MenSCs in the endometriosis pathogenesis.

The discovery of noncoding RNAs (ncRNAs), such as microRNAs, small nuclear RNAs (snRNAs), long ncRNAs (lncRNAs), and circular RNAs, has altered our understanding of the physiology and development of multiple diseases, including endometriosis (18). MiRNAs have been reported to be changed in eutopic endometrial tissue from endometriosis, eutopic versus ectopic endometrial tissue, and blood samples from women with and without endometriosis (19). Increased or reduced microRNA expression in endometriosis tissue is linked to dysregulated expression of numerous target mRNAs involved in endometriosis pathogenesis (20).

To identify a candidate miRNA for targeting endometriosis-related pathways, we employed a systematic *in silico* approach adapted from Sheikholeslami *et al*. (21). Bioinformatics tools (TargetScan, miRWalk, miRDB) were used to predict miRNAs regulating key genes up-regulated in endometriosis, affecting inflammation, proliferation, and migration (16, 22, 23). This approach ensured the selection of a novel miRNA with therapeutic potential, as detailed in the Methods section. Finally, we evaluated the effects of selected miRNA on modulating the gene expression profile and functional pattern of E-MenSCs either by direct transfection of cells or using exosomes derived from NE-MenSCs as the miRNA carrier.

## Materials and Methods

### In silico miRNA selection and the study design

*In silico* miRNA selection was done as previously described by Sheikholeslami *et al* (21). We found 15 genes based on the literature, which were up-regulated during endometriosis (16, 22, 23). They were as follows: IL-6, -8, -10, and -1β Nuclear factor kappa-light-chain-enhancer of activated B cells (NF-kb), Matrix Metalloproteinase (MMP)-2, -9, SRY-Box Transcription Factor 2 (SOX2), β-catenin, B-cell lymphoma 2 (BCL2), VEGF, IDO1, KRAS, interferon-gamma (IFN-γ), and ubiquitin specific peptidase 10 (USP10). Potential miRNAs involved in the regulation of each gene were then predicted and independently analyzed using a variety of relevant algorithms such as TargetScan (https://www.targetscan.org/vert_80/), mirWalk (http://mirwalk.umm.uni-heidelberg.de/), and miRDB (http://www.mirdb.org/) to identify the miRNA that could down-regulate the greatest number of proposed genes involved in the endometriosis. The score tables for each gene were created in Excel, and the data were merged to create a single score table in which the miRNAs were ordered by repeat rate in descending order. Finally, the most appropriate miRNA was chosen from the score table.

This experimental study was approved by the Research Ethics Committee of Islamic Azad University- Science and Research Branch (ethical code: IR.IAU.SRB.REC.1401.147). Human NE- and E-MenSCs were obtained from the cell bank established by the mesenchymal stem cell research group of the Academic Center of Education, Culture and Research (ACECR), Qom, Iran, which were preserved and identified in their laboratory (24). The samples were collected on day 2 of the menstrual cycle, corresponding to the peak of menstrual bleeding, to minimize variability due to hormonal fluctuations.

 The experimental groups were as follows:

• Group 1: E-MenSCs, which receive no treatment as the negative control (E-MenSCs)

• Group 2: NE-MenSCs, which receive no treatment as the positive control (NE-MenSCs)

• Group 3: E-MenSCs, which were transfected by the backbone vector (E-MenSCs+BB)

• Group 4: E-MenSCs, which were transfected by the miRNA vector (E-MenSCs+miR)

• Group 5: E-MenSCs, which were treated with exosomes derived from NE-MenSCs (E-MenSCs+Exo)

• Group 6: E-MenSCs, which were treated with exosomes derived from transfected NE-MenSCs by miRNA vector (E-MenSCs+T-Exo)

**Figure 1:**
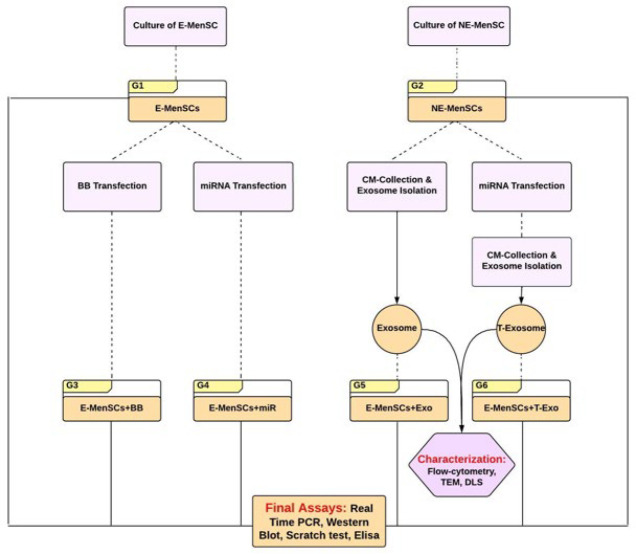
General structure of the study procedure in order to provide an overview of the experimental design, from cell culture and treatment to functional and molecular evaluations

The expression level of selected genes (Interleukin (IL)-6, -8, -10, 1β, Vascular endothelial growth factor (VEGF), β-catenin, IDO1, and KRAS, which were chosen due to selected miRNA), as well as miRNA (to confirm transfection), was tested by quantitative reverse transcription PCR (qRT-PCR). Furthermore, the level of inflammatory factors, including IL-6, -8, and Oxygen Reactive Species (ROS), was determined using the ELISA method, while the protein expression level of β-catenin and Ki-67 was determined using the western blot method. Finally, the scratching test was done to assess the migratory ability of cells in the aforementioned groups. The general structure of the study is illustrated in the flowchart below (Figure 1).

### Primer design and plasmid construction

Human bone marrow-derived MSCs were employed to extract genomic DNA. The miRNA gene was then amplified through a polymerase chain reaction (PCR) using the isolated DNA as a template. To create miRNA clones, a primer for a particular miRNA was created. After electrophoresis, the PCR product was removed from the gel. The potential miRNA gene was added to the PCDH vector. The PCR product and PCDH vector were digested using two enzymes, EcoRI and XbaI. Following ligation, the resultant product was converted into DH5á competent cells. PCR and sequencing were then used to confirm the miRNA-PCDH.

### Cell culture

Dulbecco’s Modified Eagle’s Medium (DMEM) supplemented with 10% fetal bovine serum (FBS) and 1% penicillin/streptomycin was used to culture all cells. The cultivation was performed at 37 Figure in a humidified atmosphere containing 5% CO_2_. The cells were sub-cultured until the fourth passage, when the number of cells increased (80% confluence).

### Treating cells

Despite negative and positive control groups in which E- and NE-MenSCs were cultured, respectively, without receiving any extra treatments, all other groups underwent a special treatment. For groups 3 and 4, E-MenSCs were transfected with miR or control vector, while in groups 5 and 6, the cells were treated with NE-MenSC/ miR-transfected NE-MenSC-derived exosomes. All details are as follows.

### Cell transfection

E-MenSCs were seeded in a 60*15 mm plate to be 30–60% confluent at transfection. Then, Lipofectamine™ Stem Transfection Reagent (cat#STEM00015; Invitrogen, USA) was diluted with serum-free medium in a sterile tube and gently pipetted to mix. In another sterile tube, plasmid DNA was also diluted with serum-free medium and subsequently added to the diluted Lipofectamine™ Stem Reagent (1:1 ratio). The mixture was incubated for ten minutes at room temperature, and the DNA-lipid complex was finally added to the cells in a drop-wise manner. The plate was gently shaken and swirled to ensure even distribution over the entire plate. The E-MenSCs were transfected with either the control vector (backbone) or miR-PCDH as groups 3 and 4, respectively.

*Confirming transfection*

Inverted fluorescent microscopy

Fluorescence microscopy was used to study MenSCs that had been transfected with a GFP reporter construct at 48 hr after transfection.

Real-time qRT-PCR for miRNA

Total RNA Extraction Kit (Parstoos, Iran) was used for the isolation of total RNA from the cells. The procedure was performed 72 hr after the transfection of miR-149-3p- PCDH. In order to measure miR-149-3p expression, the isolated RNA underwent a reverse transcription reaction (to obtain cDNA) using a reverse transcription PCR (RT-PCR) kit (ParsToos, Iran). The manufacturers’ instructions were followed during the whole process, except that RT primer was used instead of oligo d(T), and U6 was used as the internal control for miRNA. 2X SYBR Green Real Time PCR (ParsToos, Iran) was used for real-time PCR. The expression of miRNA was normalized using U6 as an internal control, and the 2^−ΔΔCT^ method was used to examine the relative expression levels in treated and control cells. Each test was run in triplicate. The primers’ sequences are represented in Table 1.

### CM collection and exosome isolation

The exosomes should be extracted from both NE-MenSCs and miR-transfected NE-MenSCs. For this purpose, at approximately 80% confluence (8 × 10^5^ cells/cm^2^) in the fourth passage, the media were changed and replaced with medium containing less FBS every 48 hr. After a gradual reduction of the serum level to zero, FBS-free CM was collected and passed through a 0.2 μm filter. The exosomes were isolated using the Exocib exosome extraction kit according to the manufacturer’s instructions (Cib Biotech Co) (25). In brief, an exosome precipitation solution was added to the filtered CM and incubated overnight (Figure 4). The samples were then centrifuged for 40 min at 3000 rpm, the supernatant was discarded, and the pellet was re-suspended in phosphate-buffered saline (PBS). Finally, using a Bicinchoninic Acid (BCA) protein assay kit (Sigma-Aldrich, Missouri, USA), the total protein content of the samples was measured, and the purified exosome samples were kept at 70 Figure.

*Exosome characterization*

Flow cytometry assay

Flow cytometry was performed on an FC500 flow cytometer (Beckman Coulter, Fullerton, CA, USA), and the data were processed with Beckman Coulter CXP software. Surface markers such as CD63, CD81, and CD9 were assessed in the isolated exosomes. In flow cytometry tests, all antibodies were employed at the doses recommended by the manufacturers.

Dynamic light scattering (DLS)

The DLS technique was used to measure the size distribution of the exosomes, which should be between 30 and 150 nm in diameter. Since the particles are irradiated with a laser beam in this procedure, all vesicles exposed to the beam will emit light. The fluctuations in the intensity of the diffused light will be observed, and a mathematical model based on Brownian motion and light scattering theory will be used. In brief, materials were diluted to 1 g/ml in PBS and 0.05% Tween-20, and particle size was determined using a DLS Zetasizer Nano ZS (Malvern Instruments, UK).

Transmission electron microscopy (TEM)

The exosome suspension was loaded onto a carbon-coated electron microscopy grid, followed by the addition of 2.5% glutaraldehyde in 0.1 M cacodylate buffer to the sample for two hours at 4 Figure. After 1-hour fixation with 1% osmium tetroxide in 0.1 M cacodylate buffer, the sample was rinsed three times in distilled water before being dyed for two hours at room temperature with 0.5% aqueous uranyl acetate. A JEOL 1200EX device was used to observe the samples.

*Exosome treatment of E-MenSCs*

E-MenSCs were employed in the third passage for exosome treatments, regardless of whether the exosomes were isolated from NE-MenSCs or NE-MenSCs transfected with mir-149-3p-PCDH to form groups 5 and 6, respectively. The exosomes (200 µg/ml) were added to DMEM containing 10% FBS and 1% penicillin-streptomycin per well after the cells had reached 70% confluence (17). The subsequent experiments were conducted after the cells had been cultured for 72 hr under normal conditions.

### ELISA test evaluation of inflammatory factors

Approximately 3 ×10^5^ cells/well NE- or E-MenSCs were seeded in a 6-well plate. Seventy-two hours after treatment, the expression level of IL-6, IL-8, and ROS was assessed using ELISA kit (R&D Systems; Iran) according to the manufacturer’s protocol. Briefly, in a 96-well plate, 100 μl of the assay diluent was first added to each well, followed by 100 μl of the standard, control, or sample, and incubation was allowed to occur for two hours. 200 µl of conjugated antibodies was added to each well after aspiration and washing, and incubated for two hours at room temperature. The aspiration and washing were repeated, and 200 µl substrate solution was added for 20 min in the dark, and then the stop solution was administered. Each sample’s OD was measured at 450 nm, and a standard curve was created based on the standards’ concentration. The concentration of markers in each well was calculated based on the slope of the line.

### Western blotting of β-catenin and Ki-67

For Western blot analysis of β-catenin and Ki-67, cells were lysed with Pro-PRETM cell lysis solution (iNtRON Biotechnology, Korea), and the obtained protein concentration was measured using the BCA protein assay method (iNtRON Biotechnology, Korea) and a spectrophotometer (Smartspec Plus spectrophotometer, Bio-Rad). To perform western blotting in this study, a TV100 vertical system (Scie-Plas Ltd, UK) with 10×10 cm^2^ gel units and a Consort-EV202 power generator (Sigma) was used. Briefly, 2X Laemmli sample buffer was combined in an equivalent volume with the cell lysates. After boiling for five minutes, lysates (20 g) were subjected to SDS-PAGE and then transferred to an immune-Blot^TM^ polyvinylidene difluoride (PVDF) membrane with a 0.2 μm thickness (Cat#162-017777; Bio-Rad Laboratories, CA, USA). After that, the membranes were blocked for 1 hour with 5% Bovine serum albumin (BSA) (Cat # A-7888; Sigma Aldrich, MO, USA). The membranes were then treated for 1 hour at room temperature with anti-β-catenin (Cat# ab223075, Abcam, Cambridge, UK), anti-Ki67 (Cat No. ab16667, Abcam), and anti-β-actin-loading control antibodies (Cat#ab8227, Abcam). Membranes were then incubated with goat anti-rabbit IgG H&L (HRP) secondary antibody (Cat#ab6721; Abcam) after being rinsed three times with TBST. Following this, the membranes were incubated in enhanced chemiluminescence (ECL) for 1-2 min. β-actin was used to normalize protein expression. The percentage area under the curve of each band was divided by the percentage area under the curve of its corresponding actin band as part of the densitometry of protein bands carried out using the gel analyzer Version 2010a software (NIH, USA), and the calculated values were then compared between groups as previously described (26).

### Real-time qRT-PCR for selected genes

To perform the real-time qRT-PCR for the candidate genes, the procedure described above was followed. Real-time PCR was performed 72 hr after the treatment to measure the expression level of 9 candidate genes, including IL-6, -8, -10, 1β, VEGF, β-catenin, IDO1, and KRAS. Briefly, following the manufacturer’s instructions, total RNAs were extracted using the RNeasy kit (Gene All Biotechnology, Seoul, Korea), and a Nanodrop 2000 spectrophotometer (Thermo Fisher Scientific, Wilmington, USA) was used to evaluate the purity and quantity of RNA at 260/280 nm. Applying a transcription kit (Yekta Tajhiz, Iran), single-strand cDNA was synthesized via reverse transcription. The expression of target genes was normalized using glyceraldehyde-3-phosphate dehydrogenase (GAPDH) as internal control, and the 2^−ΔΔCT^ method was used to examine the relative expression levels in treated and control cells. Each test was run in triplicate. The primer sequences of the candidate genes are represented in Table 2.

### Scratch test

The scratch assay was used to assess *in vitro* cell migration capacity when all groups were grown in 24-well plates (2×10^4^ cells/well) to full confluence. A scratch was created on the cell monolayer in the center of each well with a pipette tip, followed by washing with PBS to remove unattached cells. As a result, a cell-free zone was formed to induce cells to move and close the gap. The images were then taken at regular intervals of 0, 12, 24, and 36 hr. The wound area was determined by manually tracing the cell-free region in acquired images using ImageJ software, and the migration rate was calculated as the percentage of area reduction or wound closure in three independent samples per group using the following formula:

$\mathrm{Wound Closure\%}=\left[\frac{A_{t=0_{h}-A_{t={∆}_{h}}}}{A_{t=0_{h}}}\right]\times 100\%$

$A_{t=0_{h}}$ is the area of the wound measured immediately after scratching (t=0h)

$A_{t={∆}_{h}}$ is the area of the wound measured *h* hours after the scratch is performed

### Statistical analysis

The Kolmogorov-Smirnov test was used to confirm normal distribution of data in SPSS version 26 (SPSS, Chicago, IL, USA). Statistical analyses were performed using one-way analysis of variance (ANOVA) for most variables. For the scratch assay, two-way ANOVA was used to analyze closure percentage across treatment groups and time points. When significant differences were observed (*P*<0.05), *post hoc* pairwise comparisons were conducted using Bonferroni correction to adjust for multiple comparisons. Effect sizes were quantified using Partial Eta Squared (η²) for variables. Additionally, 95% confidence intervals (CIs) were calculated for group means and differences.

## Results

### miR-149-3p selection and cloning

**Figure 2:**
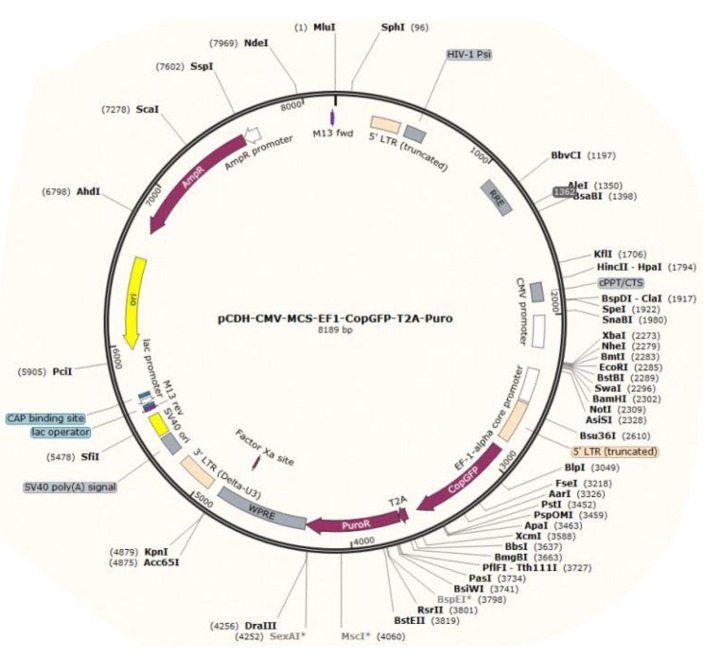
Schematic map of vector PCDH-CMV-MSC-EF1-CopGFP-T2A-Puro

To identify miRNAs regulating up-regulated genes in E-MenSCs, we used publicly accessible algorithm-based bioinformatics databases. The miRNAs were arranged in a score table from greatest to least repeat rates, and miR-149-3p - located at 2q37.3 and encoded by only one exon - was ultimately chosen. To our knowledge, no prior experimental studies have investigated miR-149-3p in endometriosis, making it a novel candidate, supported by its reported anti-inflammatory and anti-proliferative effects in cancers such as ovarian, gastric, and pancreatic cancer (27-29). Then, based on the schematic map in Figure 1, miR-149-3p was cloned into the vector PCDH, and miRNA-PCDH was purchased (Bonyakhte, Iran) (Figure 2).

### Characterization of MenSCs-derived exosomes

**Figure 3:**
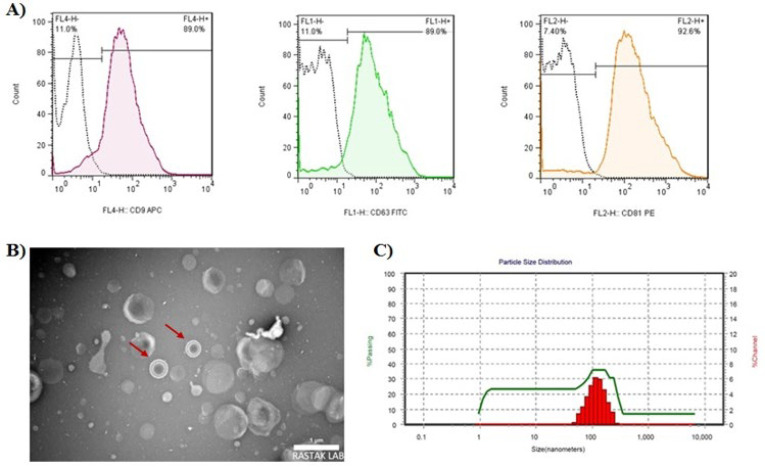
MenSCs-derived exosome characterization to validate their identity through surface marker expression by flow cytometry, morphological assessment, and particle size distribution

Utilizing flow cytometry, TEM, and DLS assays, exosomes isolated from the CM of MenSCs were characterized. According to the flow cytometry results, exosome-specific markers CD63, CD81, and CD9 were positively expressed (Figure 3A), the background of TEM images was clear and exosomes were enclosed by a bilayer lipid membrane (Figure 3B). DLS data revealed that the main particle size distributional range was between 70 and 200 nm (Figure 3C).

### Verification of cell transfection

**Figure 4:**
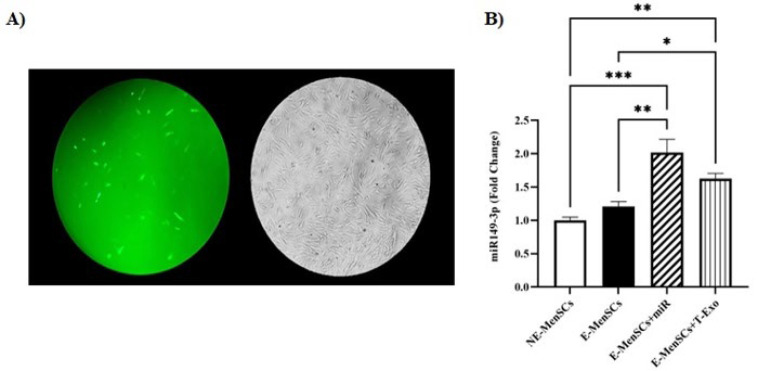
Verification of miR-149-3p-PCDH transfection through quantitative real-time PCR analysis and fluorescence microscopic observation

NE-MenSCs and E-MenSCs cell lines were cultured to around 70% confluency. The cells were then transfected with a vector containing green fluorescent protein (GFP), a fluorescent dye that could be used to determine the viability and the degree of vector entry (Figure 4A). Using real-time qRT-PCR, the entry of miR-149-3p into the cell lines was verified. As a result, transfected cells had significantly higher levels of miR149-3p (Figure 4B).

### The expression level of candidate genes using qRT-PCR

*Selected interleukin gene expression*

**Figure 5:**
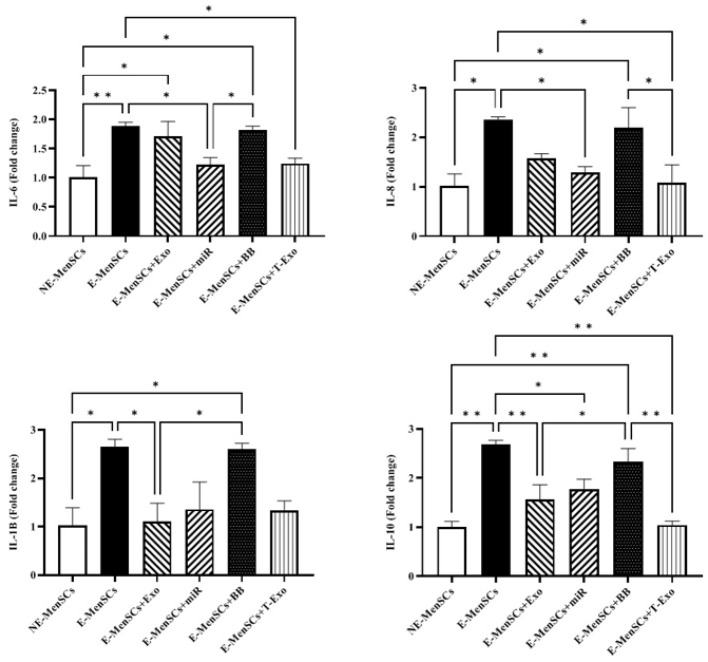
Alteration assessment of IL-6, -8, -1β, and -10 gene expression levels in different groups of experimental and control samples using qRT-PCR

Based on the obtained data shown in Figure 5, there was a significant increase in the expression level of all the assessed interleukins, including IL-6, -8, -1β, and -10, in E-MenSCs compared to the NE-MenSCs (p 0.0082, 0.0134, 0.0211, and 0.0011, respectively). In addition, E-MenSCs+Exo group showed higher IL-6 expression than the NE-MenSCs (*P*=0.0236). Similarly, the E-MenSCs+BB group was up-regulated compared to the NE-MenSC group regarding all the mentioned genes (*P*=0.0120, 0.0242, 0.0248, and 0.0038, respectively).

Moreover, IL-6, -8, and -10 genes were significantly down-regulated in E-MenSCs+T-Exo group compared to E-MenSCs (*P*=0.0364, 0.0175, 0.0012, respectively). There was also a significant decrease of IL-8 and -10 in E-MenSCs+T-Exo group rather than E-MenSCs+BB group (*P*=0.0321, and 0.0044, respectively). E-MenSCs+miR group had a significantly lower expression of IL-6, -8, and -10 to E-MenSCs, as well (*P*=0.0309, 0.0399, and 0.0239, respectively). In addition, IL-6 showed a significant decrease in E-MenSCs+miR group in comparison to E-MenSCs+BB group (*P*=0.0481). For IL-1β and IL-10, the E-MenSC+Exo group showed down regulation compared to the E-MenSC (0.0260 and 0.0089, respectively) and E-MenSC+BB (*P*=0.0306 and 0.0497, respectively) groups (Figure 5).

*VEGF and β-catenin gene expression*

**Figure 6:**
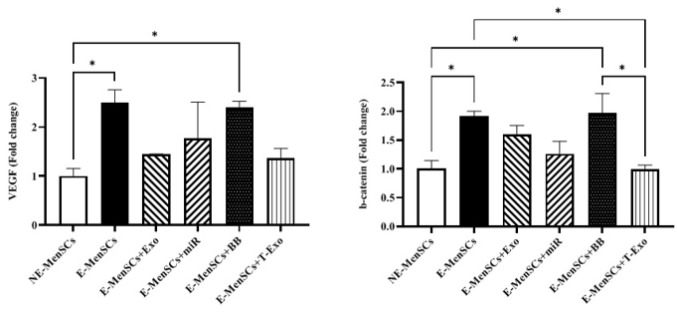
Alteration assessment of VEGF and β-catenin gene expression levels in different groups of experimental and control samples using qRT-PCR

The expression levels of VEGF and β-catenin genes were significantly lower in NE-MenSCs in comparison to E-MenSCs (*P*=0.0327 and 0.0217, respectively) and E-MenSCs+BB group (*P*=0.0453 and 0.0248, respectively). In addition, in comparison to E-MenSCs and E-MenSCs+BB groups, β-catenin expression level was significantly decreased in E-MenSCs+T-Exo (*P*=0.0205 and 0.0157, respectively) (Figure 6).

*KRAS and IDO1 gene expression*

**Figure 7:**
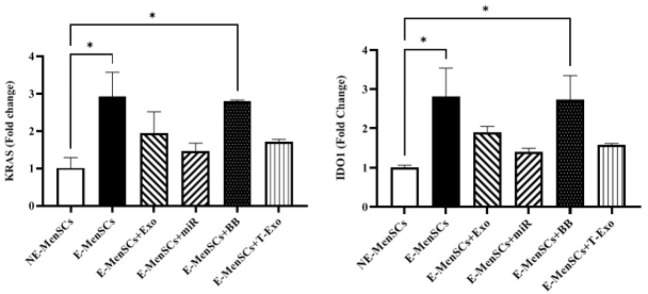
Alteration assessment of KRAS and IDO1 gene expression levels in different groups of experimental and control samples using qRT-PCR

No significant differences in KRAS and IDO1 expression were observed between treated groups and NE- or E-MenSCs, except for the E-MenSCs+BB group. However, as anticipated, KRAS and IDO1 were shown to be up-regulated in E-MenSCs and E-MenSCs+BB when compared to NE-MenSCs (*P*=0.0183 & 0.0246; *P*=0.0249 & 0.0311, respectively) (Figure 7).

### Concentration level of IL-6, -8, and ROS using the ELISA test

*IL-6 and -8 concentration*

**Figure 8:**
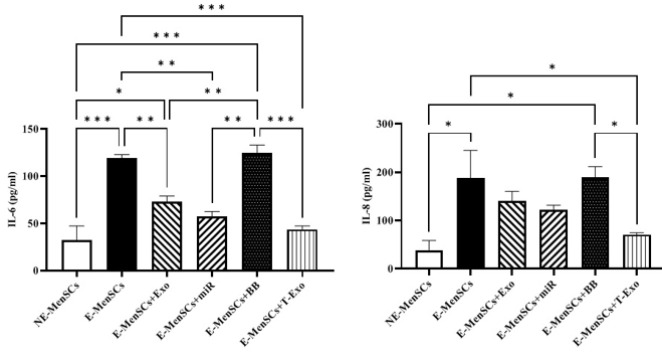
Concentration levels of IL-6 and IL-8 in different groups of experimental and control samples using the ELISA test

Both E-MenSCs and E-MenSCs+BB had greater IL-6 and IL-8 concentrations than NE-MenSCs (*P*=0.0003 and 0.0002; *P*=0.0125 and 0.0121, respectively). In contrast, IL-6 and IL-8 levels were considerably lower in the E-MenSCs+T-Exo group compared to the E-MenSCs and E-MenSCs+BB groups (*P*=0.0006 and 0.0004, respectively; 0.038 and 0.0370). In addition, E-MenSCs+Exo and E-MenSCs+miR groups had significantly lower IL-6 concentrations than E-MenSCs (*P*=0.0083 and 0.0019, respectively) and E-MenSCs+BB groups (*P*=0.0049 and 0.0012, respectively) (Figure 8). 

*ROS concentration*

**Figure 9:**
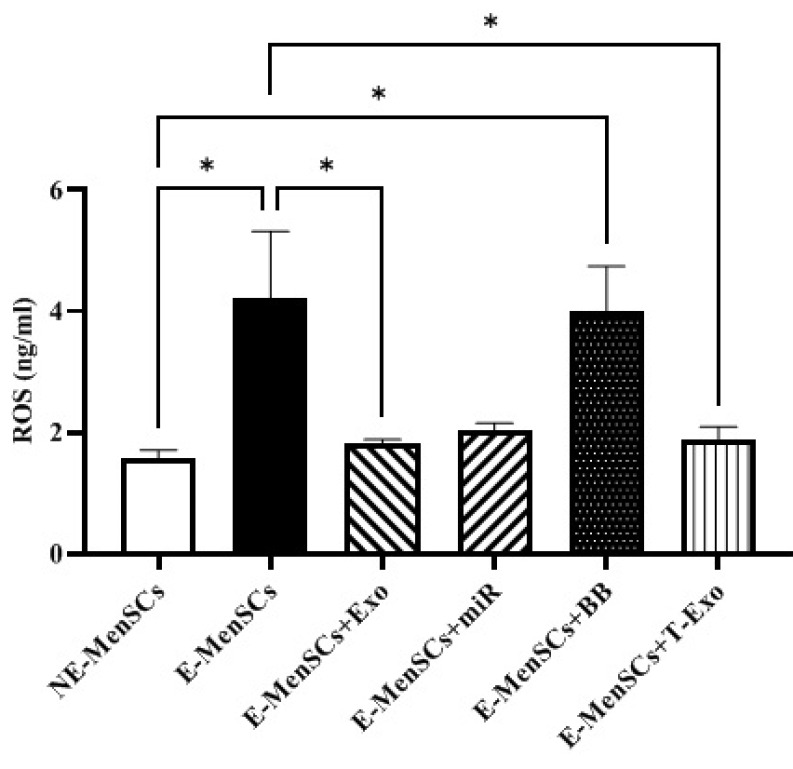
Concentration levels of ROS in different groups of experimental and control samples using the ELISA test

As shown in Figure 9, in comparison to NE-MenSCs, there was a substantial rise in ROS concentration in the E-MenSCs and E-MenSCs+BB groups when compared to NE-MenSCs (*P*=0.0226 and 0.0339, respectively). However, compared to E-MenSCs, E-MenSCs+Exo and E-MenSCs+T-Exo showed significantly lower ROS concentrations (*P*=0.0353 and 0.0397) (Figure 9).

### Protein concentration of β-catenin and Ki67 using the Western blot test

Since the levels of β-catenin in the E-MenSCs and E-MenSCs+BB groups were greater than those in the NE-MenSCs group (*P*=0.0053 and 0.0059, respectively), the shift in β-catenin protein levels in various groups was consistent with their gene expression results. Only the E-MenSCs+T-Exo group had a statistically lower protein level of β-catenin than the E-MenSCs and E-MenSCs+BB groups (*P*=0.0375 and 0.0433, respectively). 

**Figure 10:**
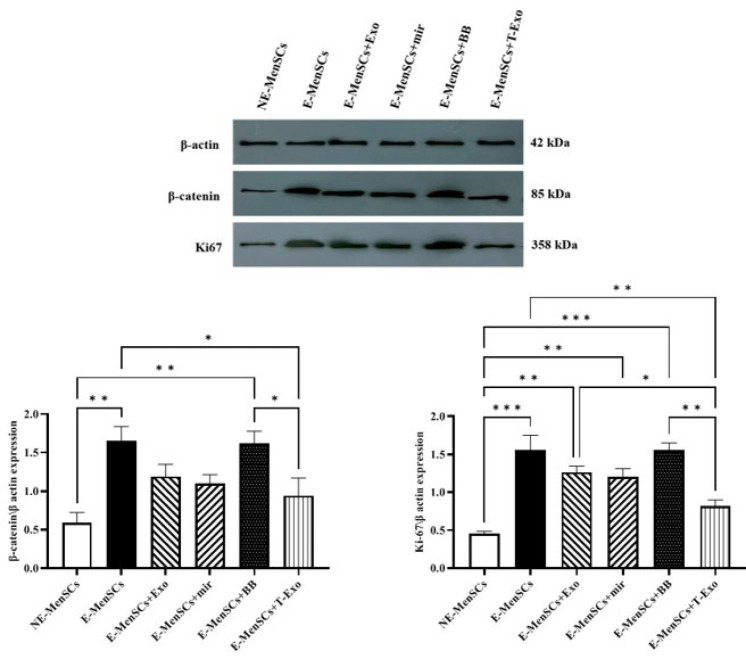
Western blot image and quantitative band density analyses of β-catenin and Ki67 in different groups of experimental and control samples

Ki67 protein levels were elevated in all groups compared to NE-MenSCs, except for the E-MenSCs+T-Exo group (*P*=0.1042). These groups included E-MenSCs (*P*=0.0004), E-MenSCs+Exo (*P*=0.0024), E-MenSCs+miR (*P*=0.0037), and E-MenSCs+BB (*P*=0.0004). Additionally, compared to the E-MenSCs, E-MenSCs+Exo, and E-MenSCs+BB groups, the E-MenSCs+T-Exo group showed a significantly lower KI67 level (*P*=0.0037, 0.0433, and 0.0038, respectively) (Figure 10). 

### Changes in migratory properties of different groups using the scratching test

**Figure 11:**
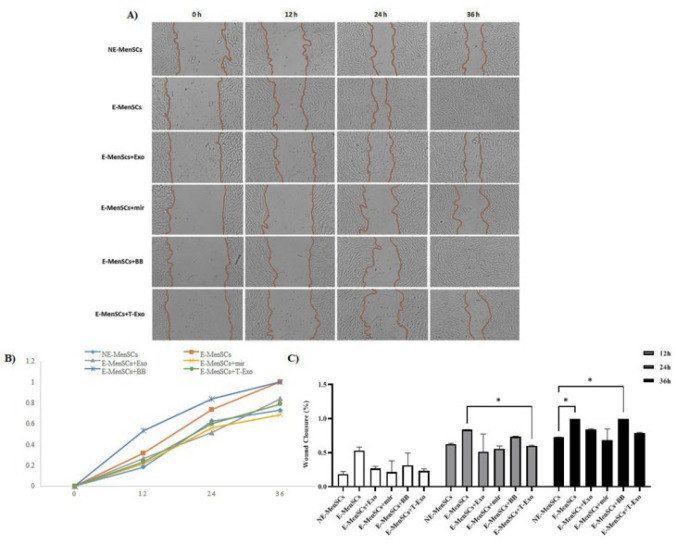
Measurement of cell migration in 12, 24, and 36 hr following the application of the scratch assay (0 hr) on MenSCs under various treatment conditions

As shown in Figures 11A and B, wound closure rates varied across groups. The capacity of the various groups to migrate was not significantly different 12 hr after scratching. At the time point of 24 hr, E-MenSCs+T-Exo group had a statistically significant lower rate of wound closure than E-MenSCs (*P*=0.0363), while after 36 hr, NE-MenSCs had only a statistically significant lower closure percentage when compared to E-MenSCs and E-MnSCs+BB groups (*P*=0.0211 for both comparisons) (Figure 11C).

## Discussion

Endometriosis significantly impairs the quality of life for women of reproductive age. Thus, developing effective treatments for this disease is crucial. Researchers have been using nanotechnology to enhance the therapeutic impact of endometriosis treatment in recent years (30). EVs, as natural nanoparticles, play crucial roles in controlling how cells function by carrying a variety of physiologically active substances, such as proteins, lipids, DNA, or mRNA. They are promising nano-carriers for drug delivery in nanomedicine due to their biocompatibility, low immunogenicity, and minimal toxicity (31, 32). Many miRNAs and lncRNAs carried by EVs have been proven to be involved in endometriosis gene therapy in recent years (32).

On the other hand, numerous studies have demonstrated that various miRNAs have an impact on endometriosis and their expression is different in the eutopic and ectopic endometria of the same endometriotic women (20). Given the differential expression of pro-inflammatory, immunological, angiogenic, cell cycle, and adhesion-related molecules in eutopic and ectopic endometrial tissues of women with endometriosis, specific miRNAs are likely to regulate gene expression. Therefore, miRNAs may be a unique and fascinating option for endometriosis diagnostic indicators and treatment targets (33).

Thus, in this study, we investigated the impact of miR-149-3p, which was chosen based on some bioinformatics databases, on modifying up-regulated target genes in E-MenSCs and various cellular functions, either through direct transfection or by treating cells with exosomes produced from NE-MenSCs transfected with miR-149-3p as miRNA carrier. 

Previous research has suggested that miR-149-3p plays a tumor-suppressive role in carcinogenesis and cancer development. For instance, a recent study found that miR-149-3p is down-regulated in ovarian cancer tissues, suppressing cell cycle arrest and apoptosis (27). Furthermore, a 2016 study found that overexpression of miR-149-3p was required for 18β-glycyrrhetinic acid-mediated suppression of cell proliferation and cell cycle progression in gastric cancer (28). In addition, by inactivating the Akt1 signaling pathway, miR-149-3p contributed to dioscin’s anti-pancreatic cancer activity (29). Previously, next-generation sequencing (NGS) and qRT-PCR were used to identify candidate miRNAs differentially expressed in endometriosis-affected women compared to healthy ones. Eight miRNAs had significantly lower circulating levels in case participants than in control subjects, including miR-199a-3p, miR-143-3p, miR-340-5p, let-7b-5p, miR-21-5p, miR-17-5p, miR-20a-5p, and miR-103a-3p (34). In this study, miR-149-3p was not differentially expressed in NE- and E-MenSCs. At the same time, there was a significantly higher mRNA expression level after cell transfection and treating E-MenSCs with T-Exo (Figure 4B).

Endometriosis is regarded as an immune-related chronic inflammatory illness because of the critical role that inflammatory immune responses play in onset and progression (35). In endometriosis, activated macrophages are significantly increased in number and produce more cytokines essential for developing lesions, proliferation, and angiogenesis. As possible treatment targets, inflammatory mediators in the pathophysiology of endometriosis have been thoroughly studied. High levels of pro-inflammatory cytokines, including IL-1, -6, -8, 33, NF-kB, and tumor necrosis factor-alpha (TNF-α), have been well documented in several endometriosis developmental stages (36). Additionally, IL-1β stimulates endometriotic cell proliferation and induces IL-6 and IL-8 production, promoting cell proliferation and inhibiting apoptosis (37). Recent studies show that sustained STAT3 activation promotes endometriosis fibrosis by enhancing IL-10 and IL-6 anti-inflammatory effects. Therefore, therapeutic approaches, such as inhibiting “inflammation,” may dysregulate the interaction between pro- and anti-inflammatory mediators, which can have negative consequences in endometriosis patients, including fibrosis (38). In this study, mRNA expression levels of all the investigated interleukins were higher in E-MenSCs and E-MenSCs+BB compared to NE-MenSCs, consistent with previous studies (16, 22). In the case of the treated groups, E-MenSCs+miR and E-MenSCs+T-Exo groups could modulate the mRNA expression level of increased IL-6, -8, -1β, and -10 of E-MenSCs. At the same time, there was no significant difference between the mentioned inflammatory factors expression level and NE-MenSCs (Figure 5). Similar to our previous study (17), E-MenSCs+Exo showed relatively the same results as miR and T-Exo treated groups, with the exception of a significantly up-regulated IL-1β in comparison to NE-MenSCs, demonstrating that treating with NE-MenSCs-derived exosomes is not as efficient as applying miR-149-3p. 

The mRNA expression level findings of IL-6 and -8 were mainly supported by their measured protein levels (Figure 8), since the amounts of IL-6 in all treated E-MenSCs were not statistically different from NE-MenSCs, except in E-MenSCs+Exo and E-MenSCs+BB, which had a significantly higher protein level of IL-6 in comparison to NE-MenSCs. Furthermore, a significantly lower IL-8 was seen just in E-MenSCs+T-Exo group compared to E-MenSCs and E-MenSCs+BB groups.

Munn *et al*. (2005) described IDO1 as an intracellular enzyme that is up-regulated during inflammation and suppresses T-cell-mediated immunological responses (39). IDO1 is expressed at the feto-maternal interface (40) and has recently been demonstrated to play a key role in the proliferation, adhesion, and invasion of endometrial stromal cells (ESCs) (41). On the other hand, it has been reported that Ras proteins play a key role in controlling the mitogenic and oncogenic activity of tyrosine kinases. Therefore, increased cell survival and proliferation are two effects of Ras activation (42). Based on our data, IDO1 and KRAS (as a part of the RAS/MAPK pathway) were up-regulated in E-MenSCs compared to NE-MenSCs. This is in line with previous studies showing higher IDO1 activity in the E-MenSCs (22) and enhanced KRAS gene expression in patients with endometriosis who have an SNP at the let-7 microRNA complementary site 6 (LCS6) in the 3′-UTR of the KRAS gene, which causes human endometrial stromal cells (hESC) to proliferate and invade more (43). However, the lack of a significant difference between any other group and NE-MenSCs represented the efficiency of applied treatments (Figure 7). Additionally, according to previous research, endometriotic tissues had higher levels of the cell proliferation marker Ki-67(44), which was consistent with our obtained results in the case of E-MenSCs. Along with this data, although E-MenSCs+Exo and E-MenSCs+miR groups had lower Ki-67 levels than E-MenSCs, they still showed significantly increased Ki-67 marker compared to NE-MenSCs. Nevertheless, the Ki-67 level of only E-MenSCs+T-Exo group was significantly decreased compared to E-MenSCs (Figure 10). These data demonstrate that T-Exo treatment most effectively reduced E-MenSCs proliferation, supported by evidence of miR-149-3p’s role in inhibiting proliferation and inducing apoptosis. MiR-149-3p reduces polo-like kinase 1 (PLK1) expression, a crucial regulator of cell cycle progression and apoptosis, through specifically targeting its 3’UTR, leading to decreased tumor cell clonogenicity and induced apoptosis (45). Additionally, caspase3,7 activity was stimulated and cell growth was markedly suppressed in neuroblastoma and HeLa cell lines transfected with human miR-149-3p (46). Furthermore, miR-149-3p was found to be increased in U87-MG glioma cells after quinidine, a voltage-gated K^+^ channel blocker therapy, which caused glioma cell proliferation to be inhibited and apoptosis to be induced (47).

Endometriosis is associated with elevated amounts of ROS and oxidative stress activity, which primarily cause cell damage and proliferation (48). Elevated ROS levels in endometriosis, driven by chronic inflammation and dysregulation of the ROS detoxification pathway, contribute to disease progression (49). It has been reported that ROS levels in endometriotic lesions were elevated by decreased catalase activity and increased hydrogen peroxide generation through mitochondrial superoxide dismutase activity (49). Hence, ROS might function as signaling molecules to maintain a proliferative phenotype linked to endometriosis. ROS act as second messengers, activating signaling pathways like extracellular signal-regulated kinase, mitogen-activated kinase, and serine/threonine protein kinase (Raf/MEK/ERK to promote cell proliferation in response to elevated endogenous ROS levels (50). In the present study, ROS level was significantly elevated in E-MenSCs compared to NE-MenSCs. However, ROS production significantly decreased in E-MenSCs treated with NE-MenSC-derived exosomes or miR-149-3p-loaded T-exosomes (Figure 9). These findings are consistent with prior work in which MSC-derived exosomes inhibited the generation of reactive oxygen species (ROS) in human skin fibroblasts caused by exposure to H_2_O_2_ and delayed oxidative stress-induced cellular senescence (51). 

Scratch assay results (Figure 11) showed significantly higher migratory ability in E-MenSCs compared to NE-MenSCs, consistent with their elevated MMP-2 and MMP-9 expression, key genes in invasion and metastasis (16). Furthermore, other researchers have shown that endometriosis tissues and cells contain higher amounts of MMPs in comparison to healthy ones, as well (52). It has been shown that up-regulated miR-149-3p inhibits the proliferation, invasion, and migration of urinary bladder cancer (BC) cells both *in vitro* and *in vivo* (53). Additionally, miR-149-3p can down-regulate the expression of S100A4, which is involved in cell differentiation, cell motility, and transcriptional regulation (54). At the same time, over-expression of S100A4 can restore the phenotypes of BC cell lines and prevent miR-149-3p’s suppressive effects (53). In this study, miR-149-3p treatment (either directly or via exosomes as a carrier) reduced the ability of E-MenSCs to migrate. Still, contrary to expectations, this reduction was not statistically significant when compared to untreated E-MenSCs.

Despite its benign nature, endometriosis exhibits malignant characteristics, such as cell proliferation, adhesion, invasion, and enhanced angiogenesis (55). These similarities may result from aberrant activation of the Wnt/β-catenin pathway, a key regulator of epithelial-mesenchymal transition (EMT) (52). We found that E-MenSCs had significantly elevated mRNA and protein expression of β-catenin (Figures 6 and 10), as well as up-regulated VEGF mRNA level in comparison to NE-MenSCs (Figure 6). Previously, the expression of β-catenin was examined in the A549 lung adenocarcinoma (LAD) cell line, when miR-149 inhibitors increased β-catenin expression; it was shown that miR-149 mimics reduced it. Therefore, it was concluded that miR-149 inhibits LAD by inhibiting the Wnt/β-catenin and EMT signaling pathways (56). According to the gene and protein assessment results, β-catenin levels may be decreased by exosome or direct transfection of miR-149-3p; however, only E-MenSCs+T-Exo demonstrated significantly lower amounts when compared to E-MenSCs (Figures 6 and 10).

Our findings suggest that miR-149-3p, delivered via exosomes, suppresses Wnt/β-catenin signaling, likely by targeting CTNNB1 or Wnt1, reducing β-catenin nuclear translocation, and potentially down-regulating EMT-related genes, which may contribute to reduced cell migration in our scratch assay (28, 56, 57). Building on the observed suppression of β-catenin by miR-149-3p-loaded exosomes, the reduction in pro-inflammatory cytokines (IL-6 and IL-8) may result from crosstalk between Wnt/β-cate nin and NF-κB signaling pathways (58). This mechanism could also contribute to the reduced migratory capacity observed in our scratch assay, despite the non-significant decrease (Figure 11). Additionally, although apoptosis was not directly assessed, miR-149-3p’s role in suppressing PLK1, a key regulator of cell cycle and apoptosis (45), may underlie the decreased Ki-67 levels (Figure 10), suggesting reduced proliferation. However, the tissue-specific complexities of endometriosis warrant further investigation to elucidate these mechanisms. Additionally, the absence of empty vector-transfected NE-MenSC-derived exosome control limits our ability to entirely exclude potential effects of the transfection process on exosome content, which should be addressed in future studies.

Our findings suggest that miR-149-3p, delivered via NE-MenSC-derived exosomes, holds promise for treating endometriosis by reducing inflammatory cytokines (Figures 5 and 8) and cellular migration (Figure 11), which may contribute to limiting endometriotic lesion growth based on their established roles in endometriosis pathogenesis (59). The exosome-based delivery system provides a biocompatible and targeted therapeutic approach, leveraging exosomes’ ability to cross biological barriers with low immunogenicity. However, clinical translation faces challenges, including targeted delivery to endometriotic lesions, *in vivo* exosome stability, and scalable production (60). Safety concerns, such as the potential off-target effects of miR-149-3p, also warrant further investigation. Future studies should prioritize *in vivo* validation of miR-149-3p in animal models of endometriosis, followed by Phase I/II clinical trials to establish safety and efficacy, paving the way for a novel, exosome-based therapy for endometriosis.

**Table 1:** Sequences of miR-149-3p primers for Real-Time PCR, presenting the forward and reverse primers applied in amplification experiments

Target	Primer	Sequence
miR-149-3p	RT	5'-GTCGTATCCAGTGCAGGGTCCGAGGT ATTCGCACTGGATACGACCAGCTG -3'
	Forward	5'-CAGAGGAAGTGGCAAAG-3'
	Reverse	5'-GTGCAGGGTCCGAGGT-3'
U6	RT	5'-GTCGTATCCAGTGCAGGGTCCGAGGT ATTCGCACTGGATACGACAATATG-3'
	Forward	5'- TACAGAGAAGATTAGCATGGC-3'
	Reverse	5'- GTGCAGGGTCCGAGGT -3'

**Table 2:** Primer sequences of selected genes, including forward and reverse strands used for quantitative Real-Time PCR analysis

Gene name	Gene bank number	Primer	Sequence (5'-3')	Product size (bp)
IL-6	NM_000600.5	F	GTGTGAAAGCAGCAAAGAGG	140
		R	CCTCAAACTCCAAAAGACCA	
IL-8	NM_001354840.3	F	GGAAGGAACCATCTCACTGT	122
		R	GTTCTTTAGCACTCCTTGGC	
IL-10	NM_000572.3	F	CCAAGACCCAGACATCAAGG	134
		R	CATTCTTCACCTGCTCCACG	
IL-1β	NM_000576.3	F	TCTTCTTCGACACATGGGATA	183
		R	GTACAAAGGACATGGAGAACA	
VEGF	NM_001171622.2	F	TGCTTGCCATTCCCCACTT	195
		R	ACTTTGCCCCTGTCGCTTT	
IDO1	NM_002164.6	F	TCCTTACTGCCAACTCTCCA	116
		R	TGTTCTCATAAGTCAGGGGC	
KRAS	NM_033360.4	F	GAGTGCCTTGACGATACAGC	142
		R	CTCCTCTTGACCTGCTGTGT	
β-Catenin	NM_001330729.2	F	GCGTGGACAATGGCTACTC	203
		R	GCCGCTTTTCTGTCTGGTT	

A) Analysis of the surface expression of CD9, CD63, and CD81 markers by flow cytometry; B) A TEM image of exosomes (red arrows show exosomes). C) Exosomes' dynamic light scattering (DLS) to determine the average size of these nanoparticles.

A) Visualization of GFP expression using fluorescence microscopy. B) qRT-PCR results showing the expression level of miR-149-3p in transfected cells compared to the negative control. *P≤0.05, **P≤0.01, ***P≤0.001.

*P≤0.05, **P≤0.01.

*P≤0.05.

*P≤0.05.

*P≤0.05, **P≤0.01, ***P≤0.001.

*P≤0.05

*P≤0.05, **P≤0.01, ***P≤0.001.

A) Sample microphotographs of wound closure in different groups. B) Quantification of the wound closure across various groups. C) Statistical analysis of wound closure percentage. *P≤0.05.

## Conclusion

This study highlights miR-149-3p as a key miRNA regulating molecular signaling pathways in endometriosis and inflammation, advancing our understanding of its biology. Exosome-encapsulated miR-149-3p, which delivers microRNAs by fusing with cell membranes, effectively modulated E-MenSCs’ function. For instance, a significant decrease in genes associated with endometriosis and inflammation, such as IL-6 and IL-8, was observed. This reduction significantly decreased cell proliferation, inflammation, angiogenesis, and migration. We conclude that exosomes, as miRNA carriers, hold potential for developing effective therapies for endometriosis.

## Data Availability

The data that support the findings of this study are available upon request from the corresponding author. The data is not publicly available due to privacy and ethical restrictions.

## Declaration

We have not used any AI tools or technologies to prepare this manuscript.

## References

[1] Taylor HS, Kotlyar AM, Flores VA (2021). Endometriosis is a chronic systemic disease: Clinical challenges and novel innovations. Lancet.

[2] Koninckx PR, Fernandes R, Ussia A, Schindler L, Wattiez A, Al-Suwaidi S (2021). Pathogenesis based diagnosis and treatment of endometriosis. Front Endocrinol.

[3] Horne AW, Missmer SA (2022). Pathophysiology, diagnosis, and management of endometriosis. Bmj.

[4] Zondervan KT, Becker CM, Missmer SA (2020). Endometriosis. N Engl J Med.

[5] Sampson JA (1927). Peritoneal endometriosis due to the menstrual dissemination of endometrial tissue into the peritoneal cavity. Am J Obstet Gynecol.

[6] Brichant G, Laraki I, Henry L, Munaut C, Nisolle M (2021). New therapeutics in endometriosis: A review of hormonal, non-Hormonal, and non-Coding RNA Treatments. Int J Mol Sci.

[7] Barra F, Ferrero S (2019). Adhesion proteins: Suitable therapeutic targets or biomarkers of therapy response for endometriosis?. Acta Obstet Gynecol Scand.

[8] Meng X, Ichim TE, Zhong J, Rogers A, Yin Z, Jackson J (2007). Endometrial regenerative cells: A novel stem cell population. J Transl Med.

[9] Dominici M, Le Blanc K, Mueller I, Slaper-Cortenbach I, Marini F, Krause D (2006). Minimal criteria for defining multipotent mesenchymal stromal cells. The International Society for Cellular Therapy position statement. Cytotherapy.

[10] Bjørge IM, Kim SY, Mano JF, Kalionis B, Chrzanowski W (2017). Extracellular vesicles, exosomes and shedding vesicles in regenerative medicine - a new paradigm for tissue repair. Biomater Sci.

[11] Maas SLN, Breakefield XO, Weaver AM (2017). Extracellular vesicles: Unique intercellular delivery vehicles. Trends Cell Biol.

[12] Lai RC, Arslan F, Lee MM, Sze NS, Choo A, Chen TS (2010). Exosome secreted by MSC reduces myocardial ischemia/reperfusion injury. Stem Cell Res.

[13] Colombo M, Raposo G, Théry C (2014). Biogenesis, secretion, and intercellular interactions of exosomes and other extracellular vesicles. Annu Rev Cell Dev Biol.

[14] Lo Cicero A, Stahl PD, Raposo G (2015). Extracellular vesicles shuffling intercellular messages: for good or for bad. Curr Opin Cell Biol.

[15] Ma ZJ, Yang JJ, Lu YB, Liu ZY, Wang XX (2020). Mesenchymal stem cell-derived exosomes: Toward cell-free therapeutic strategies in regenerative medicine. World J Stem Cells.

[16] Sahraei SS, Davoodi Asl F, Kalhor N, Sheykhhasan M, Fazaeli H, Moud SS (2022). A comparative study of gene expression in menstrual blood-derived stromal cells between endometriosis and healthy women. Biomed Res Int.

[17] Davoodi Asl F, Sahraei SS, Kalhor N, Fazaeli H, Sheykhhasan M, Soleimani Moud S (2023). Promising effects of exosomes from menstrual blood-derived mesenchymal stem cells on endometriosis. Reprod Biol.

[18] Adams BD, Parsons C, Walker L, Zhang WC, Slack FJ (2017). Targeting noncoding RNAs in disease. J Clin Invest.

[19] Santamaria X, Taylor H (2014). MicroRNA and gynecological reproductive diseases. Fertil Steril.

[20] Filigheddu N, Gregnanin I, Porporato PE, Surico D, Perego B, Galli L (2010). Differential expression of microRNAs between eutopic and ectopic endometrium in ovarian endometriosis. J Biomed Biotechnol.

[21] Sheikholeslami A, Nabiuni M, Arefian E (2017). Suppressing the molecular signaling pathways involved in inflammation and cancer in breast cancer cell lines MDA-MB-231 and MCF-7 by miR-590. Tumour Biol.

[22] Nikoo S, Ebtekar M, Jeddi-Tehrani M, Shervin A, Bozorgmehr M, Vafaei S (2014). Menstrual blood-derived stromal stem cells from women with and without endometriosis reveal different phenotypic and functional characteristics. Mol Hum Reprod.

[23] Sahin C, Mamillapalli R, Yi KW, Taylor HS (2018). microRNA Let-7b: A Novel treatment for endometriosis. J Cell Mol Med.

[24] Sahraei SS, Davoodi Asl F, Kalhor N, Sheykhhasan M, Fazaeli H, Moud SS (2022). A comparative study of gene expression in menstrual blood-derived stromal cells between endometriosis and healthy women. Biomed Res Int.

[25] Fazaeli H, Kalhor N, Naserpour L, Davoodi F, Sheykhhasan M, Hosseini SKE (2021). A comparative study on the effect of exosomes secreted by mesenchymal stem cells derived from adipose and bone marrow tissues in the treatment of osteoarthritis-induced mouse model. Biomed Res Int.

[26] Siavashi V, Nassiri SM, Rahbarghazi R, Vafaei R, Sariri R (2016). ECM-dependence of endothelial progenitor cell features. J Cell Biochem.

[27] Jiang R, Zhang H, Zhou J, Wang J, Xu Y, Zhang H (2021). Inhibition of long non-coding RNA XIST upregulates microRNA-149-3p to repress ovarian cancer cell progression. Cell Death Dis.

[28] Cao D, Jia Z, You L, Wu Y, Hou Z, Suo Y (2016). 18β-glycyrrhetinic acid suppresses gastric cancer by activation of miR-149-3p-Wnt-1 signaling. Oncotarget.

[29] Si L, Xu L, Yin L, Qi Y, Han X, Xu Y (2017). Potent effects of dioscin against pancreatic cancer via miR-149-3P-mediated inhibition of the Akt1 signalling pathway. Br J Pharmacol.

[30] Yuxue J, Ran S, Minghui F, Minjia S (2023). Applications of nanomaterials in endometriosis treatment. Front Bioeng Biotechnol.

[31] Wu P, Zhang B, Ocansey DKW, Xu W, Qian H (2021). Extracellular vesicles: A bright star of nanomedicine. Biomaterials.

[32] Wu D, Lu P, Mi X, Miao J (2018). Exosomal miR-214 from endometrial stromal cells inhibits endometriosis fibrosis. Mol Hum Reprod.

[33] Ohlsson Teague EM, Van der Hoek KH, Van der Hoek MB, Perry N, Wagaarachchi P, Robertson SA (2009). MicroRNA-regulated pathways associated with endometriosis. Mol Endocrinol.

[34] Papari E, Noruzinia M, Kashani L, Foster WG (2020). Identification of candidate microRNA markers of endometriosis with the use of next-generation sequencing and quantitative real-time polymerase chain reaction. Fertil Steril.

[35] Riccio LdGC, Santulli P, Marcellin L, Abrão MS, Batteux F, Chapron C (2018). Immunology of endometriosis. Best Pract Res Clin Obstet Gynaecol.

[36] Kang YJ, Jeung IC, Park A, Park YJ, Jung H, Kim TD (2014). An increased level of IL-6 suppresses NK cell activity in peritoneal fluid of patients with endometriosis via regulation of SHP-2 expression. Hum Reprod.

[37] Sikora J, Smycz-Kubańska M, Mielczarek-Palacz A, Kondera-Anasz Z (2017). Abnormal peritoneal regulation of chemokine activation-The role of IL-8 in pathogenesis of endometriosis. Am J Reprod Immunol.

[38] Matsuzaki S, Pouly JL, Canis M (2023). IL-10 is not anti-fibrotic but pro-fibrotic in endometriosis: IL-10 treatment of endometriotic stromal cells in vitro promotes myofibroblast proliferation and collagen type I protein expression. Hum Reprod.

[39] Munn DH, Mellor AL, Rossi M, Young JW (2005). Dendritic cells have the option to express IDO-mediated suppression or not. Blood.

[40] Jeddi-Tehrani M, Abbasi N, Dokouhaki P, Ghasemi J, Rezania S, Ostadkarampour M (2009). Indoleamine 2,3-dioxygenase is expressed in the endometrium of cycling mice throughout the oestrous cycle. J Reprod Immunol.

[41] Mei J, Jin LP, Ding D, Li MQ, Li DJ, Zhu XY (2012). Inhibition of IDO1 suppresses cyclooxygenase-2 and matrix metalloproteinase-9 expression and decreases proliferation, adhesion and invasion of endometrial stromal cells. Mol Hum Reprod.

[42] Schubbert S, Shannon K, Bollag G (2007). Hyperactive Ras in developmental disorders and cancer. Nat Rev Cancer.

[43] Grechukhina O, Petracco R, Popkhadze S, Massasa E, Paranjape T, Chan E (2012). A polymorphism in a let-7 microRNA binding site of KRAS in women with endometriosis. EMBO Mol Med.

[44] Calcagno A, Grassi T, Mariuzzi L, Marzinotto S, Londero AP, Orsaria M (2011). Expression patterns of Aurora A and B kinases, Ki-67 and the estrogen and progesterone receptors determined using an endometriosis tissue microarray model. Hum reprod.

[45] Shin CH, Lee H, Kim HR, Choi KH, Joung JG, Kim HH (2017). Regulation of PLK1 through competition between hnRNPK, miR-149-3p and miR-193b-5p. Cell Death Differ.

[46] Lin RJ, Lin YC, Yu AL (2010). miR-149* induces apoptosis by inhibiting Akt1 and E2F1 in human cancer cells. Mol Carcinog.

[47] Ru Q, Tian X, Pi MS, Chen L, Yue K, Xiong Q (2015). Voltagegated K+ channel blocker quinidine inhibits proliferation and induces apoptosis by regulating expression of microRNAs in human glioma U87MG cells. Int J Oncol.

[48] Tosti C, Pinzauti S, Santulli P, Chapron C, Petraglia F (2015). Pathogenetic mechanisms of deep infiltrating endometriosis. Reprod Sci.

[49] Ngô C, Chéreau C, Nicco C, Weill B, Chapron C, Batteux F (2009). Reactive oxygen species controls endometriosis progression. Am J Pathol.

[50] (2006). Reactive oxygen species-induced activation of the MAP kinase signaling pathways. Antioxid Redox Signal.

[51] Matsuoka T, Takanashi K, Dan K, Yamamoto K, Tomobe K, Shinozuka T (2021). Effects of mesenchymal stem cell-derived exosomes on oxidative stress responses in skin cells. Mol Biol Rep.

[52] Matsuzaki S, Darcha C (2013). Involvement of the Wnt/β-catenin signaling pathway in the cellular and molecular mechanisms of fibrosis in endometriosis. PLoS One.

[53] Yang D, Du G, Xu A, Xi X, Li D (2017). Expression of miR-149-3p inhibits proliferation, migration, and invasion of bladder cancer by targeting S100A4. Am J Cancer Res.

[54] Ebralidze A, Tulchinsky E, Grigorian M, Afanasyeva A, Senin V, Revazova E (1989). Isolation and characterization of a gene specifically expressed in different metastatic cells and whose deduced gene product has a high degree of homology to a Ca2+-binding protein family. Genes Dev.

[55] Varma R, Rollason T, Gupta JK, Maher ER (2004). Endometriosis and the neoplastic process. Reproduction.

[56] Jiang WS, Huang CL, Zhang J, Xu F, Dai XH (2020). MicroRNA-149 inhibits the progression of lung adenocarcinoma through targeting RAP1B and inactivating Wnt/β-catenin pathway. Eur Rev Med Pharmacol Sci.

[57] Valentijn AJ, Palial K, Al-Lamee H, Tempest N, Drury J, Von Zglinicki T (2013). SSEA-1 isolates human endometrial basal glandular epithelial cells: Phenotypic and functional characterization and implications in the pathogenesis of endometriosis. Hum Reprod.

[58] Ma B, Hottiger MO (2016). Crosstalk between Wnt/β-Catenin and NF-κB signaling pathway during inflammation. Front Immunol.

[59] Koh HB, Kim HJ, Kang SW, Yoo TH (2023). Exosome-based drug delivery: Translation from bench to clinic. Pharmaceutics.

[60] Palakurthi SS, Shah B, Kapre S, Charbe N, Immanuel S, Pasham S (2024). A comprehensive review of challenges and advances in exosome-based drug delivery systems. Nanoscale Adv.

